# Potential molecular mechanisms and clinical implications of piRNAs in preeclampsia: a review

**DOI:** 10.1186/s12958-024-01247-1

**Published:** 2024-06-24

**Authors:** Yuanxuan Ma, Bo Hou, Jinbao Zong, Shiguo Liu

**Affiliations:** 1 Prenatal Diagnosis Center, The Affiliated Hospital of Qingdao University, Qingdao, Shandong, 266003 Shandong China; 2 Department of Medical Genetics, the Affiliated Hospital of Qingdao University, Qingdao , Shandong, 266003 China; 3 Department of Cardiology, the Affiliated Hospital of Qingdao University, Qingdao , Shandong, 266003 China; 4 Department of Laboratory, Qingdao Hiser Hospital Affliated of Qingdao University (Oingdao Traditional Chinese Medicine Hospital), 4 Renmin Road, Qingdao, 266033 China; 5 Medical Genetic Department, the Affiliated Hospital of Qingdao University, 16 Jiangsu Road, Qingdao, 266003 China

**Keywords:** Non-coding RNA, PIWI-interacting RNA, Preeclampsia, Pregnancy, Targeted therapy, Biomarker, Epigenomics

## Abstract

Preeclampsia is a multisystem progressive condition and is one of the most serious complications of pregnancy. Owing to its unclear pathogenesis, there are no precise and effective therapeutic targets for preeclampsia, and the only available treatment strategy is to terminate the pregnancy and eliminate the clinical symptoms. In recent years, non-coding RNAs have become a hotspot in preeclampsia research and have shown promise as effective biomarkers for the early diagnosis of preeclampsia over conventional biochemical markers. PIWI-interacting RNAs, novel small non-coding RNA that interact with PIWI proteins, are involved in the pathogenesis of various diseases at the transcriptional or post-transcriptional level. However, the mechanisms underlying the role of PIWI-interacting RNAs in the pathogenesis of preeclampsia remain unclear. In this review, we discuss the findings of existing studies on PIWI-interacting RNA biogenesis, functions, and their possible roles in preeclampsia, providing novel insights into the potential application of PIWI-interacting RNAs in the early diagnosis and clinical treatment of preeclampsia.

## Background

Preeclampsia (PE) is a progressive multisystem disorder specific to pregnancy that can be divided into early-onset and late-onset phenotypes (EOPE and LOPE) based on the time of diagnosis. EOPE is commonly associated with impaired placental development and subsequent growth restriction early in pregnancy, whereas LOPE is thought to be associated with maternal endothelial dysfunction [1]. PE affects approximately 8% of pregnancies globally, with an estimated 4 million women diagnosed with PE annually, resulting in the deaths of more than 70,000 women and 500,000 fetuses and newborns [2, 3]. In the US, PE is the leading cause of maternal death, serious complications, maternal intensive care admissions, high cesarean section rates, and preterm labor. Due to scarce resources and poor access to adequate obstetric care, PE poses a more serious threat to the lives of pregnant women in low- and middle-income countries than those in high-income countries [4, 5]. However, there are currently no drugs that can prevent PE progression and the only treatment option is termination of pregnancy [4]. Therefore, there is an urgent need to develop early predictors and therapeutic targets for PE.

PIWI-interacting RNAs (piRNAs) are small non-coding RNAs with lengths ranging from 23 to 32 nt. piRNA has a 5' end uridine or tenth position adenosine preference, a 2′-O-methylation modification at the 3′ end, and is processed via a Dicer-independent mechanism from single-stranded precursor transcripts expressed in intergenic regions called piRNA clusters [6–8]. piRNAs, initially found to be highly expressed in germline cells, play important roles in germline development, stem cell self-renewal, transposon silencing, and gametogenesis in various organisms by forming complexes with PIWI proteins (PIWIL1, PIWIL2, PIWEL3 and PIWIL4) [9, 10]. In addition, piRNAs are also highly expressed in somatic cells and play regulatory roles, such as silencing transcriptional processes, regulating translation and mRNA stability, and interacting with several proteins [11]. The abundant regulatory mechanisms of piRNAs in germ cells and somatic cells have resulted in them gradually becoming a research hotspot, and researchers are increasingly exploring the mechanisms underlying the roles of piRNA in human disease pathogenesis and their promising applications in disease diagnosis and treatment. Recently, abnormal expression of some piRNAs and PIWI proteins have been observed to be involved in the pathogenesis of PE [12, 13]. Therefore, exploring the molecular mechanisms of piRNAs in PE may provide novel insights into the early prediction and treatment of PE. This review summarizes recent studies on piRNAs, including their biosynthesis, functions, possible epigenetic regulatory roles in the pathogenesis of PE, and their potential as biomarkers and therapeutic targets for PE.

## PIWI-interacting RNAs (piRNAs)

### Biogenesis of piRNAs

The motifs that give rise to piRNAs are concentrated at specific genomic loci, known as piRNA clusters [14, 15]. piRNAs can be categorized into five groups based on their origin: transposon-derived, mRNA-derived, transfer RNA (tRNA)-derived, long-stranded non-coding RNA (lncRNA)-derived, and snoRNA-derived piRNAs. Transposon-derived piRNAs originate from single-stranded clusters of transcripts, yielding both sense and antisense piRNAs. mRNA-derived piRNAs stem from the mRNA 3' untranslated region (3'UTR), such as piRNAs found in Drosophila ovarian somatic follicular cells and mouse pre-gross-lineage meiotic spermatogonia [15]. tRNA-derived piRNAs are directly produced from precursor 5'-tRNA halves (rather than mature tRNA) [16]. lncRNA-derived piRNAs, exemplified by the thick-lineage piRNAs in mouse testis, are produced from the exonic region of lncRNAs [15]. Finally, snoRNA-derived piRNAs have been observed in human CD4 primary T lymphocytes, produced through the complementary binding of pre-mRNA introns to significantly downregulate interleukin 4 (IL-4) expression, thereby inhibiting Th2 T lymphocyte development [17].

### piRNAs are generated via two major pathways

To date, although the biosynthesis of piRNAs has been extensively studied in animals such as Drosophila and mice, it has not been addressed in humans. The signature of the biogenesis of piRNAs is the transcription of piRNA precursors and their subsequent processing in the cytoplasm to produce mature piRNAs, which is a unique and conserved process [18]. piRNAs clusters are transcribed into unidirectional single-stranded transcripts, which are processed into piRNA precursors by two different mechanisms depending on the origin of the piRNA clusters [8].Subsequently, piRNA precursors translocate from the nucleus to the cytoplasm, where they undergo cleavage and modifications. This generates piRNA intermediates, which then form complexes with PIWI proteins and eventually generate mature piRNAs. This process involves two main pathways: the primary processing pathway and the "ping-pong" amplification pathway [18].

#### Primary processing pathway

Primary processing of piRNAs is dependent on the nucleic acid endonuclease Zucchini (Zuc), located in the outer mitochondrial membrane [19, 20], which generates piRNA intermediates with a strong preference for 5'U by cleavage of the 5' end of the piRNA precursors [18]. Subsequently, the 5' end of the piRNA intermediates is added to PIWI proteins and further cleaved at the 3' end, followed by 2'-O-methylation via DmHen1/Pimetmethyl transferase to generate primary piRNAs [7, 8]. After completing primary processing, some piRNA/PIWI complexes enter the nucleus to regulate target gene transcription, inducing heterochromatin formation by directing the methylation of histone 3 lysine 9 (H3K9me3), and thus silencing transposons [21, 22]. Meanwhile, the remaining complexes are involved in the next step of processing and maturation; the "ping-pong cycle".

#### The "ping-pong" amplification pathway

The "ping-pong" amplification pathway, first identified in Drosophila, is the major source of piRNAs in germ cells, combining piRNA biogenesis with piRNA-dependent post-transcriptional gene silencing (PTGS). Here, Aub-piRNA and Ago3-piRNA complexes function as nucleic acid endonucleases to cleave the piRNA precursors [23–25]. piRNAs that bind to Aub exhibit a 5'U bias signature, whereas piRNAs that bind Ago3 have an adenine at position 10, and the two types of complexes contain complementary piRNA sequences [18]. Aub cleaves the positive-sense piRNA precursors by coupling them to a piRNA of antisense strand origin (Aub-piRNA) to generate piRNA intermediates. After processing, the newly generated piRNA (positive strand origin) is loaded onto Ago3 and acts as a slicer to cleave antisense piRNA precursors, thereby generating another piRNA (antisense strand origin), which is loaded onto Aub for subsequent rounds of amplification [26, 27].

### Biological functions of piRNAs

In germ cells, piRNAs silence transposon activity and gene expression in a sequence-dependent manner to promote fertility and ensure genome integrity [28–31]. Moreover, recent evidence suggests that piRNAs are also expressed in many human tissues in a tissue-specific manner, but the extent of their expression and their roles in somatic cells remain unknown. piRNAs may also be involved in regulating the pathogenesis of various diseases (cardiovascular, neurological, respiratory, and urological diseases and various cancers) at the transcriptional or post-transcriptional level [27, 32–37].

#### piRNA/PIWI complex-mediated transcriptional gene silencing (TGS)

piRNA-mediated gene silencing depends on the subcellular localization of the PIWI or piRNA-induced silencing complex (piRISC). Transcription regulation is primarily carried out by PIWI proteins located in the nucleus (e.g. Drosophila PIWI and mouse MIWI2) [38]. piRNA/PIWI mediates TGS by inhibiting mRNA transcription through histone modification and de novo methylation machinery. The piRNA/PIWI complex binds to Asterix protein and enters the nucleus, where it forms a new complex after binding to Panoramix, the only known TGS-specific factor capable of inducing repression when artificially attached to chromatin or extended transcripts. Then, silencing mechanism components are recruited to initiate TGS [6, 27, 32]. First, lysine-specific demethylase 1 removes the activated histone 3 lysine 4 dimethylation (H3K4me2) marker from the promoter region, inhibiting transcription by RNA Pol II. Then, the repressive histone 3 lysine 9 trimethylation (H3K9me3) marker is recruited to the target DNA by Eggless and its cofactor Windei [27, 39]. Subsequently, heterochromatin protein 1 is recruited, leading to heterochromatin formation and transcription repression [24]. In addition, piRNAs and PIWI proteins alter transcriptional activity by regulating DNA methyltransferase (DNMT), which promotes methylation of CpG islands in the promoter region [39].

#### piRNA/PIWI complex-mediated post-transcriptional gene silencing (PTGS)

PTGS is carried out by the cytoplasmic piRISC through a mechanism similar to that of RNA interference (RNAi), by cleaving target mRNAs [40, 41]. For example, in Drosophila embryos, Aub directly binds to the CDS and 3′ UTR of maternal mRNAs to promote their decay in a piRNA-dependent manner [42]. The PIWI proteins involved in this event are Drosophila Aub and mouse Mili (Piwil2), both of which have nuclease endonuclease (slicer) activity similar to that of Drosophila Ago2, which cleaves RNA [38]. piR-001773 and piR-017184 have recently been shown to bind to PIWIL4 to form piRNA/PIWI complexes and promote prostate cancer (PCa) cell proliferation, migration, and invasion by mediating *PCDH9* mRNA degradation through an miRNA-like mechanism in PCa cells [43]. In addition to mRNAs, piRNAs can also interact with lncRNAs [26, 44] and pseudogenes [45]. Cardiac apoptosis-associated piRNA (HAAPIR) promotes cardiomyocyte apoptosis by recruiting N-acetyltransferase 10 (NAT10) to facilitate the acetylation of *TFEC* mRNA and thus is a key regulator of apoptosis in cardiomyocyte cells in response to ischemia and reperfusion injury [46].

#### piRNA/PIWI complex-mediated protein modification

In addition to mediating TGS of target genes, the PAZ structural domain of the piRNA or PIWI proteins in piRNA/PIWI complexes can directly bind to certain proteins. piR-L-163, whose expression is downregulated in non-small cell lung cancer, plays a key role in ERM activation by directly binding to the phosphorylated ERM protein [31]. In colorectal cancer (CRC), piR-54265 specifically binds to PIWIL2 and promotes the formation of the PIWIL2/STAT3/phospho-SRC complex and phosphorylation of STAT3, thereby promoting the proliferation and metastasis of CRC cells. In this process, PIWIL2 directly recruits STAT3 through the PAZ structural domain [47].

## piRNAs: potential epigenetic regulators of PE pathogenesis

**Fig. 1:**
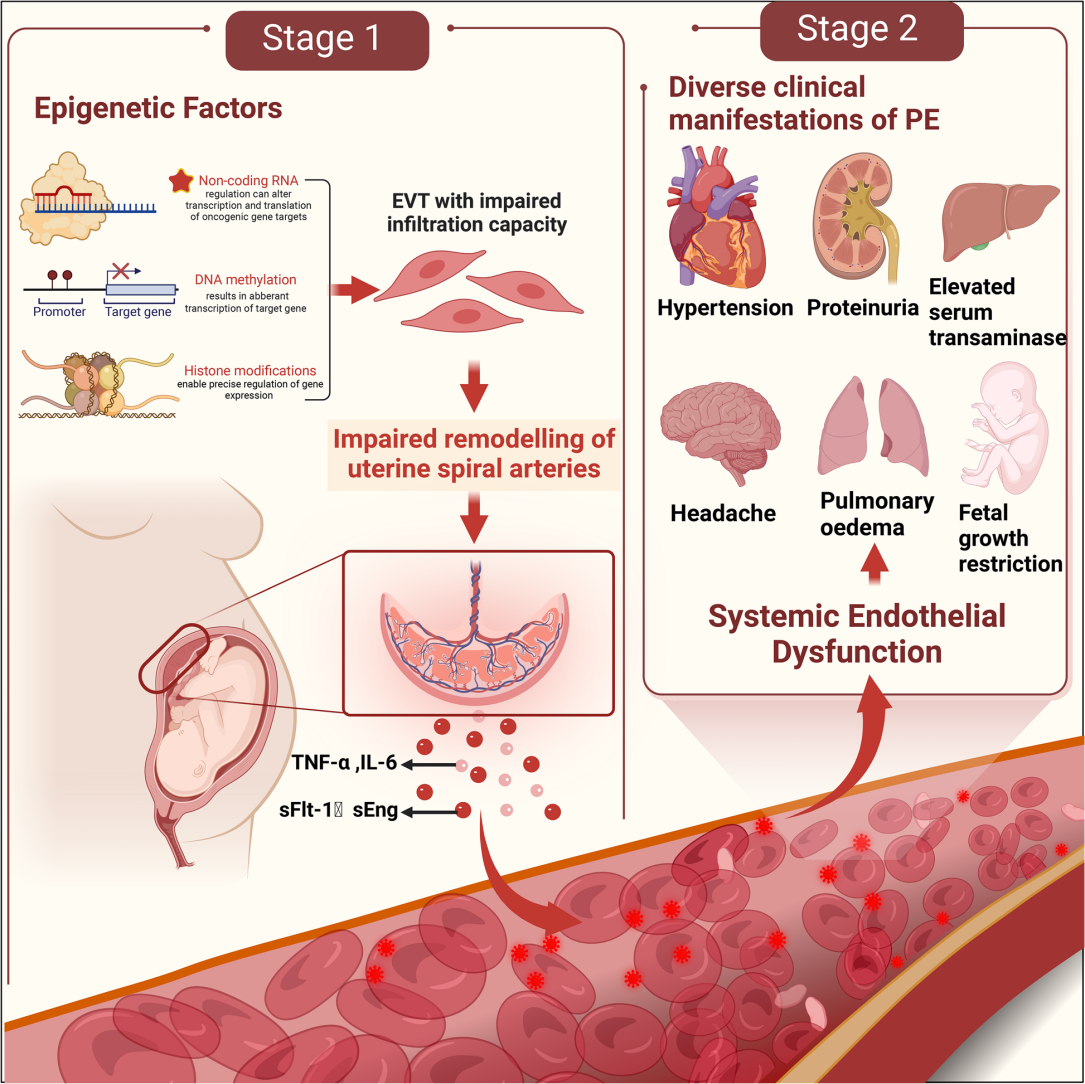
Epigenetic factors in the pathogenesis of PE. Epigenetic factors including non-coding RNA, DNA methylation and histone modification trigger an impaired EVT infiltration capacity, leading to dysfunctional remodelling of the uterine spiral arteries, causing placental hypoxia–ischemia and the release of excessive anti-angiogenic factors (sFlt-1,sEng) and inflammatory factors (IL-6,TNF-α), which is the first stage in the pathogenesis of PE. Subsequently, excessive placental factors enter the maternal circulation, promoting the activation of systemic inflammatory responses and extensive frontal vascular endothelial damage, ultimately causing a reduction in the perfusion of systemic organs, which is harmful to the mother and child, which is the second stage of the pathogenesis of PE. sFlt-1:Soluble Fms-Like Tyrosine Kinase-1, sEng:solubleendoglin, EVT: extravillous trophoblast, IL-6: interleukin-6, TNF-α: tumor necrosis factor-α, PE: preeclampsia

The pathogenesis of PE includes inadequate remodeling of the uterine spiral arteries, inflammatory immune hyperactivation, vascular endothelial cell injury, genetic factors, and insulin resistance [48]. Impaired remodeling of the uterine spiral arteries is widely recognized as the underlying cause of PE development [49]. In the early stages of normal pregnancies, extravillous trophoblast (EVT) cells infiltrate the inner 1/3 of the myometrium and enter the lumen of small uterine spiral arterioles, gradually replacing smooth muscle cells and elastin in the vessel wall, transforming the arterioles into low-resistance high-capacity blood vessels to increase placental blood flow and ensure adequate oxygen and nutrition delivery to the fetus [50]. However, in PE, EVT cells cannot enter the lumen of small uterine spiral arterioles. In addition, EVT cells fail to invade the meconium and myometrium, and the ensuing inadequate perfusion and high velocity, turbulent blood flow originating from poorly remodeled spiral arterioles leads to placental ischemia–reperfusion injury and oxidative stress, which is the first stage in the pathogenesis of PE [4]. This process is believed to be associated with impaired smooth muscle replacement in the vascular wall of spiral arteries and atherosclerosis [51]. In the second stage, the damaged placenta releases anti-angiogenic, pro-inflammatory, and other deleterious factors into the maternal circulation, leading to activation of the inflammatory response as well as endothelial dysfunction, which ultimately causes diverse clinical manifestations [5]. This is the widely recognized two-stage theory of the pathogenesis of PE, in which epigenetic regulation mediated by non-coding RNAs plays a crucial role (Fig. 1).

### Role of non-coding RNA-mediated epigenetics in preeclampsia etiology

A large number of epigenetic alterations, such as altered DNA methylation, non-coding RNA regulation, and histone modifications, are present in the preeclamptic placenta and other affected tissues, which may play an important role in the progression of the disease [52]. Among them, non-coding RNAs regulate gene expression and pathogenesis. Many non-coding RNAs have been shown to be aberrantly expressed in the placentas or blood vessels of patients with PE, and they promote placental development by regulating the expression of genes associated with trophoblast cell proliferation and invasion; activating the NO-NOS pathway to regulate vascular resistance and hemodynamic changes [53]; or targeting angiogenic factors to promote angiogenesis [54]. miR-145-5p mediates trophoblast cell invasion in female patients with EOPE by regulating TNF expression [55].Zhang et al. found that lncRNA DANCR activates the PI3K/AKT pathway through downregulation of miR-214-5p expression, promoting chorionic trophoblast cell migration and invasion of chorionic trophoblasts [56]. Zhao et al. found that lncRNA FEZF1-AS1 regulates apoptosis and proliferation of placental trophoblasts by stabilizing NOC1L mRNA expression through interaction with ELAVL2 [57]. In addition, miR-574-5p and miR-1972 may act as anti-angiogenic factors that inhibit endothelial cell proliferation and migration and reduce the ability of endothelial cells to form angioid structures by downregulating the expression of the cell proliferation marker MKI67 [58]. Dysregulation of any of these regulatory non-coding RNAs may lead to the development of PE. Recent studies have shown that piRNAs have also been observed in placental tissues. Martinez et.al first described the transcriptome of piRNAs in the human placenta, they identified more than 200 piRNAs preferentially expressed in the human placenta, with a large portion of placental piRNAs being expressed from a single locus, among them, the 15 most highly expressed placental piRNAs mapped to the *DLK1-DIO3* locus, all of which suggest that piRNAs may play specific roles in the placenta through complex mechanisms such as gene-specific regulation and mediation of placenta-specific epigenetic regulation [59]. Interestingly, researchers have recently identified the presence of differentially expressed piRNAs in PE placenta [12]. piRNAs that are potential causative agents in PE pathogenesis are shown in Table 1.

**Table 1:** piRNAs as potential causative agents in the first stage of PE pathogenesis

piRNAs	Targets	Possible functions	Reference
uniq_277797	FXR1	uniq_277797 indirectly regulates PIWIL1 protein expression by modulating *FXR1*, inhibits proliferation, migration and invasion, and promotes apoptosis in trophoblast cells	[12]
uniq_271431	DDX6	uniq_271431 inhibits proliferation, migration and invasion and promotes apoptosis of trophoblast cells by regulating DDX6 to inhibit PIWIL1 protein expression	[12]
piR-55490	Akt/mTOR signaling pathway	piR-55490 Inhibits cell proliferation by binding to the 3'UTR of mTOR mRNA and inducing its degradation to inhibit the activation of the Akt/mTOR pathway in trophoblast cells	[102]
piR-39980	RRM2	piR-39980 targets the 3'UTR of *RRM2* and may inhibit trophoblast cells proliferation and induce apoptosis by reducing RRM2 expression	[132]
piRNA-MW557525	p53 signaling pathway	piRNA-MW557525 promotes NB cell apoptosis by activating the p53 signaling pathway to induce G0/G1 phase block in trophoblast cells	[86]
piR-36712	SEPW1P	piR-36712 acts directly on *SEPW1P* RNA and indirectly activates p53 by reducing its expression and stability, thereby inhibiting the ability of trophoblast cells to proliferate, migrate and invade	[45]
piR-63049	Wnt2B	piR-63049 inhibits Wnt/β-cateninsignaling pathway activity by degrading *Wnt2B* mRNA thereby inhibiting trophoblast cells formation	[98]
piR-162725	MAPK signaling pathway、 TGF-β signaling pathway	piR-162725 has potential regulatory effects on MAPK signaling pathway and TGF-βsignaling pathway in trophoblast cells	[95]
piR-19166	CTTN、MMPs signaling pathway	piR-19166 significantly impedes trophoblast cells migration by targeting the CTTN /MMPs pathway	[99]
piR-33151	ADAMTS6、NDST1、DNM2 SNX17	piR-33151 promotes an increase in vascular resistance leading to progressive vascular remodelling, triggering an increase in blood pressure	[101]
piR-63076	Acadm	piR-63076 inhibits Acadm expression by inducing methylation of the *Acadm* promoter region, leading to abnormally elevated pulmonary artery vascular resistance	[133]
piR-55490	mTOR signaling pathway	piR-55490 promotes angiogenesis by regulating the mTOR signalling pathway	[102]
piRNA-823	G6PD、HIF-1α signaling pathway	piR-823 reduces intracellular ROS content by enhancing G6PD expression and upregulating HIF-1α expression to create a hypoxic environment causing vascular injury	[105]
piR_004506	IL-6	piR-004506 mediates the aberrant expression of IL-6 by targeting the exonic region of IL-6 mRNA and, causing a vascular inflammatory response	[107]
piR-34393 piR-38240	CYCS、KPNA6	Low expression of CYCS and Karyopherin α6 caused by piR-34393 and piR-38240 produce excess free radicals and affect cellular homeostasis in oxidative stress state	[109]
piR-31470	GSTP1	piR-31470 inhibits the expression of GSTP1 by inducing its hypermethylation in a direct or indirect manner, triggering cellular inflammatory responses and oxidative damage	[110]

### Facilitators of impaired remodeling of the spiral arteries of the uterus

**Fig. 2:**
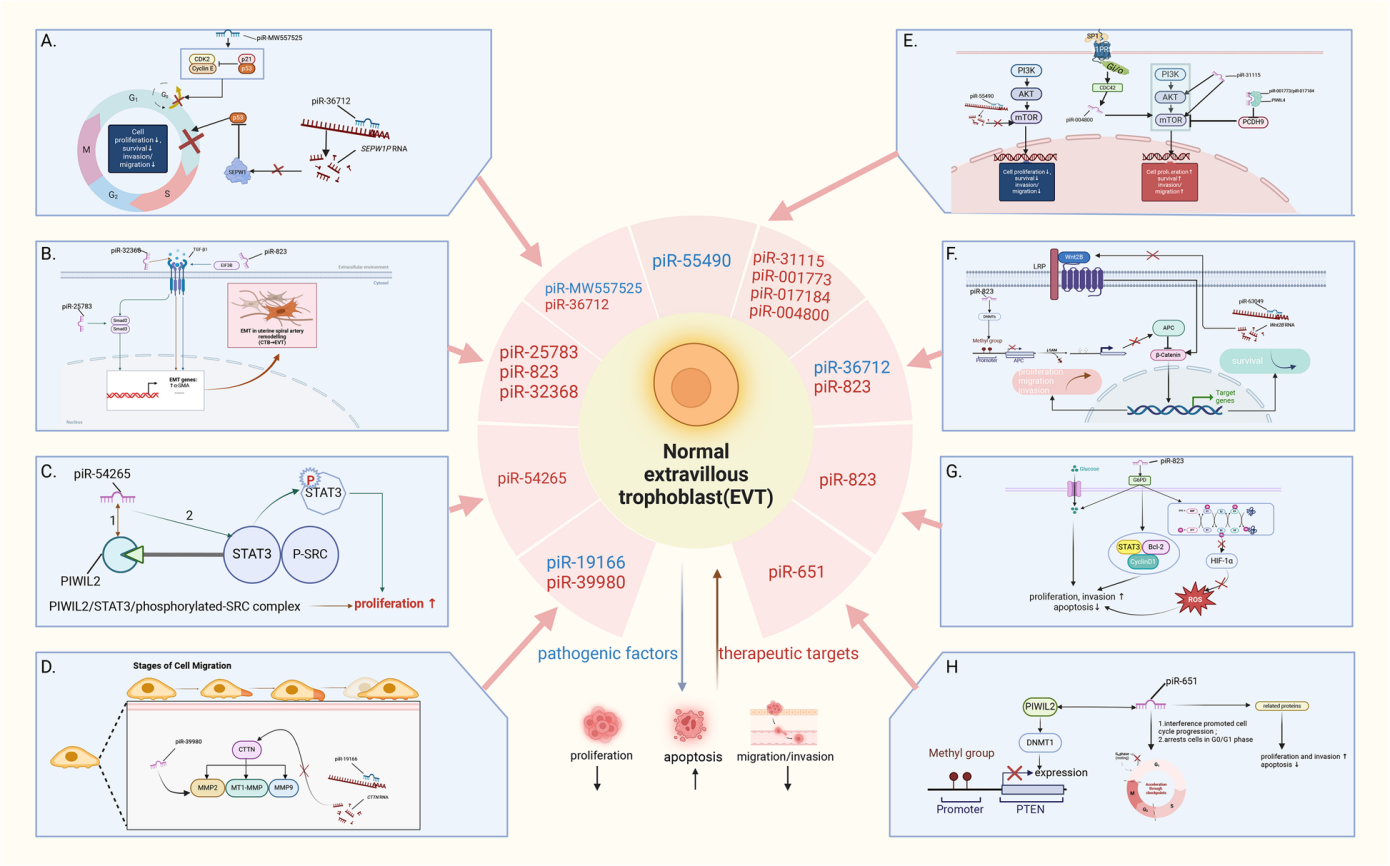
Potential pathogenic factors and therapeutic targets in stage I. The piRNAs that inhibit EVT proliferation, migration and invasion or promote its apoptosis (blue) may be potential causative factors targeting the first stage of PE pathogenesis, as they may inhibit the infiltrative capacity of EVT and thus lead to impaired remodelling of the spiral arteries of the uterus, and conversely, the piRNAs that play a positive role in the generation of EVT with a normal infiltrative capacity (red) may be the therapeutic A. ~ H. indicate specific targets or pathways in which piRNAs potentially promote or inhibit the infiltrative capacity of EVT, respectively. A. p53 signaling pathway B. TGFβ signaling pathway C. STAT3 signaling pathway D. CTTN /MMPs signaling pathway E.PI3K/AKT/mTOR signaling pathway F. Wnt/β-catenin signaling pathway G. G6PD/HIF-1αsignaling pathway H. DNMT1. p21: tumor protein21, p53: tumor protein53, CDK2: cyclin dependent kinase 2, SEPW1P: selenoprotein W pseudogene 1, SEPW1: selenoprotein W, 1, TGF-β1: transforming growth factor-β1, EIF3B: eukaryotic translation initiation factor 3 subunit B, Smad2/3: SMAD family member 2/3 CTB: Cytotrophoblast cell, EMT: Epithelial-Mesenchymal Transition, α-SMA: alpha smooth muscle Actin, STAT3: signal transducer and activator of transcription 3, CTTN: cortactin, MMP2/9: matrix metallopeptidase 2/9, MT1: metallothionein 1, PI3K/AKT/mTOR: phosphatidylinositol 3 kinase/protein kinase B/mammalian target of rapamycin, S1P: SITE-1 protease, S1PRS: sphingosine-1-phosphate receptors, CDC42: cell division cycle 42, PCDH9: protocadherin 9, Wnt2B:Wnt family member 2B, G6PD: glucose-6-phosphate dehydrogenase, ROS: reactive oxygen species, HIF-1α: hypoxia inducible factor, DNMT1: DNA methyltransferase 1, PTEN: phosphatase and tensin homolog

Impaired remodeling of uterine spiral arteries due to defective trophoblast infiltrative capacity during early embryo implantation underlies the pathogenesis of PE [48]. Abnormalities in the expression of related factors can affect the expression of signaling pathways or key factors, thereby altering the normal biological behavior of trophoblasts (Fig. 2). Using high-throughput sequencing, Lin et al. detected three piRNAs (piR-1256314, uniq_277797, uniq_271431) with upregulated expression in PE placentas, which are associated with the mTOR, apelin, autophagy, and Wingles (Wnt) signaling pathways. Further, the above piRNAs inhibit the expression of PIWIL1 protein in placental trophoblasts by regulating *FXR1* and *DDX6* expression, thereby negatively affecting the proliferation, migration, and invasion ability of trophoblasts and promoting apoptosis, which ultimately causes superficial placental attachment leading to the onset of PE [12]. He et al. detected differentially expressed piRNAs in PE placentas, including piR-4153, piR-8488, piR-15254, piR-16926, piR-16984, piR-20364, and piR-23338, and concluded that differentially expressed piRNAs regulate ECM formation and remodeling through miRNA-like PTGS, indirectly regulating trophoblast invasion. In addition, the activity of differentially expressed piRNAs in the pathogenesis of PE may occur via the phosphoinositide 3-kinase (PI3K)/Akt signaling pathway [13].

The PI3K/Akt signaling pathway is widely present in cells and plays an important role in cell survival, metastasis, metabolism, and angiogenesis [60–62]. Its dysregulation is involved in the pathogenesis of various diseases, such as osteoarthritis [63], neurological dysfunction [64, 65], diabetes [66], male infertility [67], cancer and others [68–73]. Several abnormal cellular behaviors were observed in trophoblasts following the inhibition of PI3K/Akt signaling [74–79]. Therefore, piRNAs that inhibit this pathway may disrupt the normal function of trophoblasts, thereby leading to PE. Peng et al. reported that piR-55490 inhibits the activation of the Akt/mTOR pathway in lung cancer cells by binding to the 3'UTR of *mTOR* mRNA and inducing its degradation, thereby inhibiting cell proliferation [80]. Another important pathway associated with PE is the p53 pathway, which is a key regulator of cell division, DNA replication, and the cell cycle [81]. p53 is activated when a cell is stressed or damaged beyond repair and inhibits cell proliferation or induces apoptosis, and it is overexpressed in the placenta and serum of patients with PE [82, 83]. As important factors in the p53 signaling pathway, non-coding RNA may act in the pathogenesis of PE through the regulation of the p53 pathway. Bao et al. reported that circ-DMNT1 regulates the JAK/STAT signaling pathway to promote trophoblast apoptosis by directly binding to the p53 protein and upregulating its expression, which arrests the cell cycle and inhibits cell invasion [81]. Alternatively, miR-495 restricts HDAC2 (an epigenetic p53 regulator) expression, activating the p53/PUMA axis, thereby inhibiting trophoblast proliferation, invasion, and migration and promoting apoptosis [84]. piRNAs have complementary binding sequences for p53, and differentially expressed piRNAs have been identified in p53 mutants [85]. As piRNAs have very similar functions and expression patterns to those of miRNAs, it is reasonable to assume that piRNAs can inhibit cell proliferation and promote apoptosis by regulating the p53 pathway, thereby promoting apoptosis and thus participating in the pathogenesis of PE. In fact, Tao et al. found that piR-MW557525 inhibited the G0/G1 phase in neuroblastoma (NB) cells by activating the p53 signaling pathway, which promoted apoptosis in NB cells [86]. Tan et al. suggested that piR-36712 has an indirect role in regulating the p53 pathway as it binds the pseudogene *SEPW1P* RNA and reduces the expression and stability of *SEPW1P* RNA. Subsequently, the expression of SEPW1, which is regulated by *SEPW1P*, is inhibited to further upregulate p53 expression, thereby impeding the G1 cell cycle, and ultimately reducing the proliferation, invasion, and migration of breast cancer cells [45].

In addition, there are several signal transduction pathways and related factors that regulate human trophoblast invasion/migration, such as the mitogen-activated protein kinase (MAPK) signaling pathway [87–89]、Wnt signaling pathway [90–92] and matrix metalloproteinases (MMPs) [93, 94] which are associated with the impaired remodeling of spiral arteries and are regulated by the complementary binding of piRNAs [95–97]. For example, piR-63049 has a 7 nt putative binding site containing for Wnt2B 3'-UTR, which negatively regulates Wnt/β-catenin pathway activity by inhibiting the expression of Wnt2B, a highly conserved member of the Wnt family, thereby inhibiting osteoclastogenesis in bone marrow stromal cells [98]. In addition, piR-19166 significantly impedes the migration of PCa cells by targeting the CTTN /MMPs pathway [99].

### Facilitators of vascular damage

**Fig. 3:**
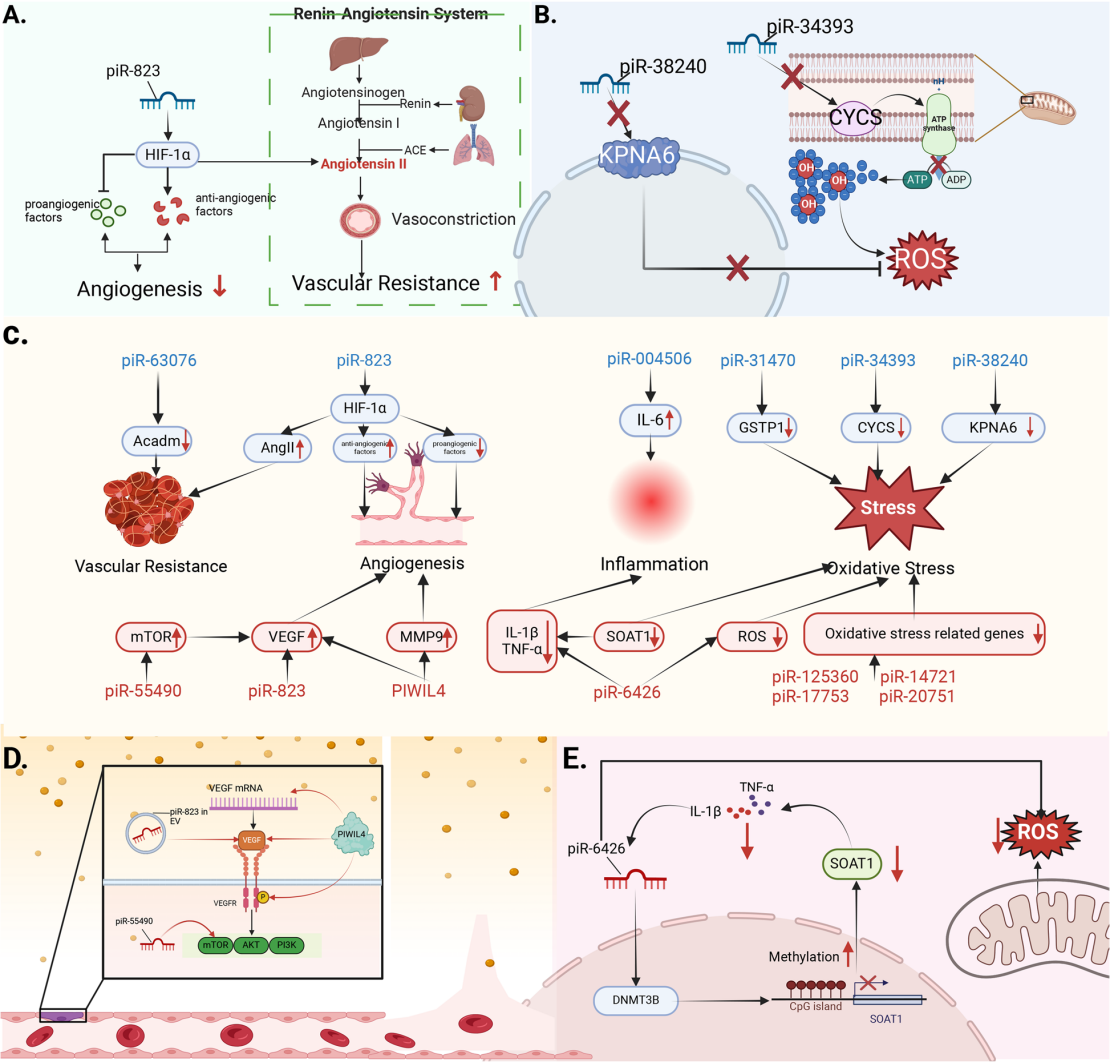
Potential pathogenic factors and therapeutic targets in stage II. The piRNAs that promote the enhancement of vascular resistance, inhibition of angiogenesis, induction of inflammatory response and oxidative stress (blue) may be potential pathogenic factors targeting the second stage of PE pathogenesis, and, conversely, the piRNAs that inhibit the above four processes (red) may be therapeutic targets for this stage. A. piR-823 triggers anti-angiogenic factors and Angiotensin II overproduction through overexpression of HIF-1α leading to increased vascular resistance as well as suppressed angiogenesis. B. piR-38240 and piR-34393 induce oxidative stress by generating excess ROS. C. Potential causative factors and therapeutic targets in the second phase of PE pathogenesis and their downstream targets or pathways are summarised. D. Intravesicular piR-823, piR-55490, and PIWIL4 promote VEGF secretion in different ways to induce angiogenesis. E. piR-6426 inhibits the expression of SOAT1 (Suppressor of Inflammatory Factor Release) by promoting its methylation which in turn leads to the overproduction of IL-1β and TNF-α triggering an inflammatory response, and induces oxidative stress by promoting ROS overproduction. KNPA6: karyopherinα6, CYCS: cytochrome c somatic, ADP: adenosine diphosphate, ATP: adenosine triphosphate, ROS: reactive oxygen species, Acadm: acyl‐CoA dehydrogenase, AngII: angiotensin II, HIF-1α: hypoxia inducible factor, GSTP1: glutathione s-transferase Pi 1, mTOR: mammalian target of rapamycin, VEGF: vascular endothelial growth factor, MMP9: matrix metalloproteinase-9, IL-1β:interleukin- 1β, TNF-α: tumor necrosis factor, SOAT1: sterol o-acyltransferase 1, VEGFR: vascular endothelial growth factor receptor, AKT: protein kinase B, PI3K: phosphatidylinositol 3 kinase

Excessive placental antiangiogenic factor (soluble fms-like tyrosine kinase 1, sFlt1) antagonizes vascular endothelial growth factor (VEGF) and placental growth factor (PlGF), thereby inducing oxidative stress, lipid peroxidation, endothelial dysfunction, and peripheral vasoconstriction during the second phase of PE pathogenesis [100]. In normal pregnancy, maternal vascular resistance is reduced, resulting in decreased blood pressure, while in PE, these functions are disrupted due to systemic vascular disease and endothelial dysfunction, which ultimately manifests in the cardiovascular and urinary systems as hypertension and proteinuria [100]. Therefore, we propose that piRNAs associated with angiogenesis and vascular injury may act as regulators in the second stage of PE pathogenesis (Fig. 3). In a study of chronic thromboembolic pulmonary hypertension, 21 aberrant piRNAs, including piR-33151, were identified as regulatory factors of four target genes, namely, *ADAMTS6, NDST1, DNM2, and SNX17*, which are crucial for vascular development and promote increased vascular resistance and thus, progressive vascular remodeling, thereby elevating blood pressure [101]. This suggests that piRNAs are associated with the development of vascular tissue remodeling. In addition, piR-55490, a regulator of the mTOR pathway, promotes angiogenesis and cardiac repair and tissue regeneration, which further indicates the potential regulatory role of piRNAs in angiogenesis [102].

Hypoxia-inducible factor (HIF), consisting of HIF-α and HIF-β subunits, is a hypoxia-activated compensatory pro-angiogenic factor [103]. HIF-α is localized in the cytoplasm and is the only oxygen-regulated subunit that determines the activity of HIF-1. HIF-1α can induce the expression of anti-angiogenic factors and inhibits the expression of pro-angiogenic factors. In addition, HIF-1α induces renin angiotensin system (RAS) expression by increasing circulating angiotensin II (AngII), leading to endothelial dysfunction and peripheral vasoconstriction, which in turn triggers PE [104]. piR-823 regulates the glucose-6-phosphate dehydrogenase (G6PD)/HIF-1α pathway by enhancing the expression of the key downstream signaling molecule G6PD, thereby inhibiting the ubiquitination of HIF-α and up-regulating the expression of HIF-α, prolonging its half-life, and reducing the intracellular ROS content to regulate the hypoxic environment [105]. Therefore, it is reasonable to assume that piR-823 triggers an increase in vascular resistance and causes vascular damage, leading to increased blood pressure by promoting the expression of HIF-α.

Increasing evidence suggests that the activation of the immune system and inflammation are key steps in vascular injury in PE [103]. Pro-inflammatory cytokines, such as IL-6, are important mediators of the maternal immune system, which are over-secreted by maternal immune cells in PE, and they activate and destroy endothelial cells by increasing vascular permeability [106]. Recent studies have shown that piR-004506 induces aberrant IL-6 mRNA expression by targeting its exonic region [107].

Oxidative stress is another important mechanism in endothelial dysfunction and vascular injury, defined as an imbalance between pro-oxidants and antioxidants. In PE, this imbalance results in extensive endothelial cell oxidative damage [108]. piR-34393 and piR-38240 inhibit the expression of cytochrome C somatic (CYCS) and KPNA6 encoding *karyopherin α6*, both of which are associated with cellular oxidative stress. Dysfunction of CYCS impairs mitochondrial ATP production, resulting in the generation of free radicals in mitochondria, whereas karyopherin maintains cellular homeostasis during oxidative stress [109]. Glutathione S-transferase pi 1 (GSTP1) is a protective gene that attenuates inflammatory responses, reduces cellular ROS production, and repairs oxidative damage. The PIWIL4/piR-31470 complex can cause cellular oxidative damage by methylating the promoter region of *GSTP1* mRNA to modulate its expression at the epigenetic level [110].

#### piRNAs: potential therapeutic targets for PE

piRNAs with potential as therapeutic targets for PE are shown in Table 2.piRNAs that modulate pathways that promote trophoblast proliferation, migration, and invasion may be potential therapeutic targets for the first stage of PE pathogenesis. The PI3K/AKT signaling pathway is a target of many piRNAs, therefore these piRNAs have the potential to restore the normal function of PE placental trophoblasts through activation of the PI3K/AKT signaling pathway [80, 97].Du et al. observed that low expression of piR-31115 inhibits clear cell renal carcinoma (ccRCC) cell proliferation, invasion, and migration, and the phosphorylation levels of AKT and PI3K were significantly reduced after knockdown of piR-31115, suggesting that piR-31115 may contribute to the promotion of relevant cellular functions through activation of the PI3K/AKT pathway [111]. In addition, piR-001773 and piR-017184 can bind to PIWIL4 to form PIWI/piRNA complexes that inhibit the expression of PCDH9, an inhibitor of the PI3K/AKT pathway [43]. Furthermore, piR-004800 expression is regulated by the S1PR signaling pathway, which can prevent apoptosis by regulating the PI3K/Akt/mTOR pathway [112].

**Table 2:** piRNAs as potential therapeutic targets for PE

piRNAs	Targets	Possible functions	Reference
piR-31115	PI3K/AKT signaling pathway	piRNA-31115 promotes trophoblast cells proliferation, migration and invasion through activation of the PI3K/AKT pathway	[111]
piR-004800	PI3K/AKT signaling pathway	Regulated by the S1PR signalling pathway, piR-004800 indirectly regulates the PI3K/Akt/mTOR pathway and thereby restrains apoptosis in trophoblast cells	[112]
piR-25783	SMAD2、SMAD3	piR-25783 activates the TGF-β/SMAD2/SMAD3 pathway to enhance trophoblast cells proliferation and metastasis	[115]
piR-32364	TGF-β1 signaling pathway	piR-32364 may potentially regulate the TGF-β signaling pathway in trophoblast cells	[117]
piR-823	EIF3B*、APC* 、G6PD/HIF-1α、VEGF	piR-823 promotes trophoblast cells proliferation, migration and invasion by activating the Wnt signalling pathway via methylating the APC promoter,or TGF-βsignalling pathway by upregulating TGF-β1 expression at the post-translational level by binding to EIF3B protein in trophoblast cells, or regulating the G6PD/HIF-1α pathway. Or promotes VEGF secretion for angiogenic activity	[105, 116, 118, 121]
piR-001773 piR-017184	PCDH9	piR-001773 and piR-017184 bind to PIWIL4 to form a complex that inhibits PCDH9 expression and indirectly promotes trophoblast cells growth。	[43]
piR-651	DNMT1	piR-651 promotes proliferation and migration and induces apoptosis in trophoblast cells by promoting DNMT1-mediated methylation of the *PTEN* promoter	[134]
piR-39980	MMP-2	piR-39980 Promotes migration and invasion of trophoblast cells by promoting MMP-2 activation	[119]
piR-54265	PIWIL2	piR-54265 binds to PIWIL2 protein to form the PIWIL2/STAT3/p-SRC complex, which activates STAT3 signalling and promotes the proliferation and metastasis of trophoblast	[47]
piR-6426	inflammatory factors (IL-1βTNF-α) 、ROS; DNMT3B	piR-6426 inhibits secretion of inflammatory factors and ROS production or ameliorates dysfunction of vascular endothelial cells with oxidative stress-induced injury by promoting SOAT1 promoter methylation through recruitment of DNMT3B	[125]
piR-125360 piR-14721 piR-17753 piR-20751	PIWIL1/PIWIL2	These piRNAs degrade the mRNAs of oxidative stress-related genes by binding to PIWIL1/PIWIL2 to form a silencing complex and thus reduce the vascular damage caused by oxidative stress	[126]

Epithelial-mesenchymal transition (EMT) is a key mechanism for the differentiation of human cytotrophoblasts (CTBs) into EVTs, and disruption of this mechanism leads to the generation of EVTs with insufficient invasive capacity to complete EMT to effectively achieve maternal spiral artery remodeling, which in turn triggers PE [113]. The TGF-β pathway regulates EMT and is modulated by piRNAs [114]. For example, piR-25783 activates the TGF-β/Smad2/Smad3 pathway in fibroblasts by increasing Smad2 and Smad3 phosphorylation, promoting the fibroblast-to-myofibroblast transition (FMT) process and enhancing the proliferative and metastatic properties of tumor cells [115]. In addition, piR-823 up-regulates TGF-β1 expression at the post-translational level by binding to EIF3B protein [116]. Furthermore, piR-32364 may potentially regulate the TGF-β pathway [117].

In addition, piRNAs that activate the Wnt signaling pathway are also potential therapeutic targets. piR-823 indirectly activates the Wnt signaling pathway by methylating the adenomatous polyposis coli (APC) promoter and promotes cell proliferation, migration, and invasion [118]. In addition, piR-823 inhibits ubiquitination of HIF-6α by up-regulating glucose-1-phosphate dehydrogenase (G1PD) expression and ultimately up-regulates glucose consumption and inhibits intracellular ROS levels in cancer cells. Thus, piR-823 may promote proliferation, invasion, and anti-apoptosis of CRC cells by regulating the G6PD/HIF-1α pathway [105]. PiR-39980 promotes migration and invasion of the human osteosarcoma cell line HOS by facilitating MMP-2 activation [119]. piR-54265 binds to the PIWIL2 protein to form the PIWIL2/STAT3/phosphorylated-SRC (p-SRC) complex, which activates STAT3 signaling and promotes proliferation and metastasis in CRC cells [47].

In the second stage of PE pathogenesis, placental factors enter the maternal circulation and promote vascular endothelial cell injury, activating the systemic inflammatory response and resulting in clinical manifestations such as hypertension and proteinuria. Thus, we believe that piRNAs that promote angiogenesis or play roles in vascular injury restoration are promising therapeutic targets for PE. The VEGF family play crucial roles in the complex and coordinated vascular and angiogenic processes under physiological and pathological conditions. VEGF is known for its endothelial vasodilatory effects mediated through NO formation and prostacyclin release, and defects in this signaling pathway during PE result in endothelial dysfunction, proteinuria, and hypertension [120]. The existence of binding sites for piRNAs on VEGF and their possible regulation of the VEGF pathway are suggestive of the role of piRNAs in regulating the pathogenesis of diseases associated with angiogenesis disorder. piR-823 in extracellular vesicles translocates to the endothelium to promote the secretion of VEGF, thus enhancing angiogenic activity [121]. Since many piRNAs need to bind to PIWI proteins to form complexes in order to fulfil their biological functions, the possible role of PIWI proteins in neovascularization should not be overlooked. VEGF binds to the VEGF receptor (VEGFR) and activates the VEGFR tyrosine kinase, which promotes endothelial cell proliferation, migration, and tube formation. Knockdown of PIWIL4 inhibits VEGF secretion and inhibits the phosphorylation of VEGFR2, which in turn inhibits angiogenesis [122]. This regulatory role has been confirmed in studies of diabetic retinopathy (DR). In addition, PIWIL4 has been shown to regulate the expression of MMP 9, which is important for neoangiogenesis [123, 124]. Hong et al. found that piR-6426 inhibits the secretion of inflammatory factors (IL-1βand TNF-α) as well as the production of ROS. In addition, piR-6426 promotes SOAT1 (which promotes the release of inflammatory factors) methylation by recruiting DNA-methyltransferase 3B (DNMT3B) to ameliorate oxidative stress-induced dysfunction in damaged cells [125].Knockdown of glutathione peroxidase 5 (GPX5), a sperm oxidative damage protector, resulted in a significant decrease in the expression of piRNAs (piR-125360, piR-14721, piR-17753, and piR-20751) that bind to PIWIL1/PIWIL2. The mRNA levels of their target genes were up-regulated, most of which were oxidative stress-related genes [126]. This suggests that these piRNAs degrade the mRNA of oxidative stress response genes, thereby reducing oxidative damage by binding to PIWIL1/PIWIL2 to form a silencing complex.

#### piRNAs: peripheral blood biomarkers for early prediction of PE

Abnormal expression of piRNA influences the pathogenesis of PE, exemplifying the their potential as valuable diagnostic biomarkers. Researchers have demonstrated the differential expression of piRNAs in the placentas of patients with PE [12, 13], however, the level of piRNAs in PE serum, which is more easily sampled, efficiently monitored, and longitudinally assessed for therapeutic dynamics, has not been studied. piRNAs can readily cross cell membranes into the circulation, and are stable in blood and resistant to degradation by ribonucleases in bodily fluids. In addition, their levels can be easily and reliably measured by quantitative real-time polymerase chain reaction; therefore, they can be used for routine processing of clinical samples [39, 127].

Mai et al. assayed serum piR-54265 levels under different storage conditions, including immediate detection by fresh extraction, incubation at 37 °C for 24 h, maintenance at room temperature for 72 h, and repeated freezing and thawing, and no significant changes were detected [128]. This provides strong evidence that piRNAs are stable in serum. Gál et al. Identified piR-016658 as an early biomarker of preterm PE from plasma exosomes samples of pregnant women in early pregnancy by the next generation sequencing and qPCR [129]. However, the small sample size is a noteworthy issue and further validation in more patients is still needed. In addition, the investigators pointed out that due to the limitations of blood sample size and difficulty of exosome extraction, directly obtaining piR-016658 from blood samples or other body fluids may be more appropriate. However, piRNA as a noninvasive biomarker of PE has been little studied in peripheral blood. Nevertheless, piRNAs have been extensively studied as serum tumor biomarkers. For example, piR-823 is the standard biomarker for screening circulating tumor cells (CTCs) in gastric cancer, positively correlates with T stage and distant metastasis, and has a higher positive detection rate compared with that of conventional markers such as serum carcinoembryonic antigen (CEA) and carbohydrate antigen 19–9 (CA19-9) [39]. An increased risk of lymph node metastasis was associated with higher levels of piR-823, and piR-823 showed high specificity (AUC = 0.713) for detecting esophageal squamous cell carcinoma (ESCC), suggesting its potential as a diagnostic and prognostic biomarker. In addition, piR-13643 and piR-21238 expression correlates with the clinical stages of papillary thyroid carcinoma (PTC), and they show better specificity than the current biomarker, HBME1, in differentiating between benign and malignant nodules [130]. Additionally, two piRNAs have been found to represent the molecular signature related to hypertension-associated urinary albumin excretion (UAE). piR-32157 expression is up-regulated in the urinary and plasma exosomes of hypertensive patients and down-regulated in plasma. piRNA-33056 expression was up-regulated in plasma exosomes and plasma but down-regulated in the urinary exosomes of patients with UAE [131].

Collectively, these findings reflect the great potential of piRNAs as diagnostic biomarkers. However, the relatively low expression levels of piRNAs in bodily fluids compared with those of other small RNAs, such as miRNAs, may hinder their detection and analysis. In addition, since EOPE and LOPE have different pathogenesis and the role piRNAs play in the pathogenesis of PE may be related to their expression levels, there may be significant differences in the expression of piRNAs in the placenta and maternal circulation of different subtypes of PE. This emphasizes that the homogeneity of the patient population should be emphasized in the study of piRNAs as noninvasive biomarkers of PE. Therefore, the application of piRNAs as non-invasive biomarkers for diagnosing diseases remains difficult.

## Conclusions and perspectives

In this review, by summarizing the piRNAs that regulate PI3K/Akt/mTOR, p53 signaling pathway, MAPK and other signaling pathways that may play a promotional or inhibitory role in uterine helical artery remodeling disorders、vascular endothelial cell damage and activation of inflammatory response, we thoroughly studied the functions of piRNAs and their regulatory mechanisms on PE pathogenesis, and searched for stable early screening molecular markers and new diagnostic and therapeutic targets in peripheral blood of patients with PE, which are of great significance for the early diagnosis and treatment of PE. To the best of our knowledge, this is the first review describing the possible roles of piRNAs in PE pathogenesis and as early diagnostic factors and therapeutic targets for PE.

However, as mentioned above, the same piRNAs may play opposite roles in the pathogenesis of PE, such as piR-823. Therefore, more studies are needed to investigate the specific mechanisms by which piRNAs play a role in PE pathogenesis. For example, in vitro and in vivo functional assays should be performed to investigate at what level piRNAs interact with target genes or pathways.

Currently, with the development of next-generation sequencing and other advanced detection technologies, as well as the stability of piRNAs in peripheral blood, detecting aberrant expression of piRNAs in the peripheral blood of PE patients has become possible. However, the reliability of using piRNAs for PE diagnosis remains unclear. There have been no studies on the expression levels of piRNAs in PE peripheral blood, and whether they differ from that of healthy pregnant women remains unknown. Although piRNAs are differentially expressed in PE, a defined threshold for piRNA expression levels in PE and physiological blood pressure elevation during pregnancy is required. In addition, whether piRNAs are expressed at different levels at different stages of pregnancy also requires investigation. Finally, the higher cost of detecting and identifying piRNAs in tissues or bodily fluids compared with that of existing assays limits the widespread use of piRNAs as biomarkers.

Based on the summary of the existing findings, our next priority is to investigate whether differentially expressed piRNAs are present in the peripheral blood of patients with PE, whether they are PE-specific, and whether their activity is interfered with by other pathological or physiological factors. In addition, the differences in piRNA expression between PE and normal pregnancy will be further investigated to determine the optimal gestational week for early prediction of PE. After completion of the above work, in vitro experiments will be conducted to investigate the roles of differentially expressed piRNAs in trophoblasts and vascular endothelial cells to determine the specific mechanisms involved in the pathogenesis of PE, which will ensure the rationality and scientific validity of the use of piRNAs as biomarkers of PE in peripheral blood and lay the foundation for the application of piRNAs as therapeutic targets for the early treatment of PE. Overall, we hope that the advances described in this review will stimulate further research on piRNAs as non-invasive early predictors and therapeutic targets for PE.

## Data Availability

No datasets were generated or analysed during the current study.

## Abbreviations

PE: Preeclampsia
piRNA: PIWI-interacting RNA
tRNA: Transfer RNA
lncRNA: Long-stranded non-coding RNA
3'UTR: 3' Untranslated region
IL-4: Interleukin 4
Zuc: Zucchini
H3K9me3: Histone 3 lysine 9
PTGS: Post-transcriptional gene silencing
piRISC: PIWI or piRNA-induced silencing complex
H3K4me2: Histone 3 lysine 4 dimethylation
DNMT: DNA methyltransferase
PCa: Promote prostate cancer
CRC: Colorectal cancer
EVT: Extravillous trophoblast
Wnt: Wingles
PI3K/AKT/mTOR: Phosphatidylinositol 3 kinase/protein kinase B/mammalian target of rapamycin
NB: Neuroblastoma
MAPK: Mitogen-activated protein kinase (
VEGF: Vascular endothelial growth factor
HIF: Hypoxia-inducible factor
AngII: Angiotensin II
sFlt-1: Soluble Fms-Like Tyrosine Kinase-1
sEng: Solubleendoglin
IL-6: Interleukin-6
TNF-α: Tumor necrosis factor-α
p21: Tumor protein21
p53: Tumor protein53
CDK2: Cyclin dependent kinase 2
SEPW1P: Selenoprotein W pseudogene 1
TGF-β1: Transforming growth factor-β1
EIF3B: Eukaryotic translation initiation factor 3 subunit B
Smad2/3: SMAD family member 2/3
CTB: Cytotrophoblast cell
EMT: Epithelial-Mesenchymal Transition
α-SMA: Alpha smooth muscle Actin
STAT3: Signal transducer and activator of transcription 3
CTTN: Cortactin
MMP2/9: Matrix metallopeptidase 2/9
MT1: Metallothionein 1, S1P: SITE-1 protease, S1PRS: sphingosine-1-phosphate receptors
CDC42: Cell division cycle 42
PCDH9: Protocadherin 9
G6PD: Glucose-6-phosphate dehydrogenase
ROS: Reactive oxygen species
HIF-1α: Hypoxia inducible factor
PTEN: Phosphatase and tensin homolog
KNPA6: Karyopherinα6
CYCS: Cytochrome c somatic
ADP: Adenosine diphosphate
ATP: Adenosine triphosphate
Acadm: Acyl‐CoA dehydrogenas
